# Risk of infertility following pelvic angiographic embolization in female patients with pelvic fractures: A nationwide population-based cohort study in Taiwan

**DOI:** 10.1371/journal.pone.0174733

**Published:** 2017-12-01

**Authors:** Sheng-Der Hsu, Shih-Yu Lee, Kuen-Tze Lin, Chun-Shu Lin, Wu-Chien Chien, Cheng-Jueng Chen, Chi-Hsiang Chung, Wei-Kuo Chang

**Affiliations:** 1 Division of Traumatic and General Surgery, Department of Surgery, Tri-Service General Hospital, National Defense Medical Center, Taipei, Taiwan; 2 Graduate Institute of Aerospace and Undersea Medicine, National Defense Medical Center, Taipei, Taiwan; 3 Department of Radiation Oncology, Tri-Service General Hospital, National Defense Medical Center, Taipei, Taiwan; 4 School of Public Health, National Defense Medical Center, Taipei, Taiwan; 5 Department of Medical Research, Tri-Service General Hospital, National Defense Medical Center, Taipei, Taiwan; 6 Division of Gastroenterology, Department of Internal Medicine, Tri-Service General Hospital, National Defense Medical Center, Taipei, Taiwan; University of Toledo, UNITED STATES

## Abstract

Pelvic angiographic embolization is an effective procedure to provide haemostasis in patients with pelvic fractures. However, management with repeated follow-up radiographs may result in infertility. The study aimed to evaluate the risk of infertility following pelvic fracture treated with pelvic angiographic embolization in female patients. We used data from the National Health Insurance Research Database (NHIRD) provided by the Bureau of National Health Insurance of the Department of Health in Taiwan from the period of 1997–2010. A total of 36 and 18,029 patients were included in the case and control cohorts, respectively. The risk estimations for the case and control cohorts were compared using a Cox’s proportional hazards regression model. The significance level was set at <0.05. After adjusting for possible confounding factors, the incidence of infertility in the case cohort was nearly 30.7-fold higher than that in the control cohort (adjust hazard ratio [HR] = 30.7, 95% confidence interval [CI] = 10.643–70.109). Patients between 15–35 years of age had a much higher incidence of infertility in the case cohort than in the control cohort (adjusted HR = 49.9, 95% CI = 15.177–64.099). Taken together, pelvic fractures in female patients treated with arterioembolization for haemostasis might be associated with a higher risk of infertility in Taiwan. Physicians should be aware of the link and inform patients of this risk prior to arterioembolization.

## Introduction

Among patients with multiple blunt traumatic injuries, 5%–16% sustain trauma to the pelvic ring, resulting in a mortality rate of 11%–54%, primarily due to haemorrhagic shock [1–3]. Rapid haemostasis is necessary to avoid the “lethal triad” of hypothermia, coagulopathy, and acidosis secondary to hypotension and tissue hypoperfusion [4].

Embolization is commonly used to control arterial bleeding after pelvic trauma. Selective angiogram with injection of each hypogastric artery is recommended. However, the potential for distal bleeding from collateral circulation can be a significant problem. In patients who are haemodynamically unstable with multiple areas of contrast extravasation, nonselective proximal embolization of the anterior, posterior, or occasionally the hypogastric artery with Gelfoam (Pfizer, New York, USA) can be a lifesaving procedure.

Serious complications have been reported after pelvic angiographic embolization (PAE) including uterine and bladder necrosis, paresis, buttock ischaemia, and impotence [5–7]. In addition, many researchers have evaluated the relationship between PAE for postpartum haemorrhage and fertility. They concluded that embolization is a safe and effective non-surgical method that does not alter subsequent fertility [8,9]. However, based on our observation and clinical experience, radiation exposure in these patients is high due to repeated follow-up radiographic exams after PAE. The association between PAE in pelvic fractures and infertility is currently under investigation.

The definition of infertility is failure of a couple to conceive after one year of normal regular intercourse without contraception in women less than 35 years of age or after six months of normal regular intercourse without contraception in women 35 years and older [10]. Unexplained infertility accounts for 15% of female infertility [11,12].

In this study, we hypothesized that, due to radiation exposure, infertility may be increased in female patients with pelvic fracture treated with PAE. Therefore, we performed a large-scale nationwide retrospective cohort study using the Taiwan National Health Insurance Research Database (NHIRD) to evaluate the risk of infertility following PAE in female pelvic fracture patients.

## Methods

### Data source

Taiwan began its National Health Insurance (NHI) program, a single-payer and universal insurance plan, with 97% coverage rate among clinics and hospitals in 1996. Almost 99% of the population of Taiwan received health care provided by the NHI [13] in 1998. The NHI created the NHIRD for researchers in Taiwan, which has been widely applied in epidemiologic and clinical studies [14–16]. The NHIRD includes the annual registration files and original claims data for reimbursement, and is managed by the National Health Research Institutes (NHRI).

We used data from the NHIRD, provided by the Bureau of National Health Insurance of the Department of Health in Taiwan, from the period of 1997–2010. In this study, we collected disease histories from inpatient registry files of the NHIRD. Personal identification information is encrypted and unavailable before the data are released for research in order to protect patient privacy. The disease diagnoses were acquired from the International Classification of Diseases, Ninth Revision, Clinical Modification (ICD–9–CM).

### Study population

Our study was approved by the Institutional Review Board II of the Tri-Service General Hospital, National Defense Medical Center (approval number 1-105-05-050). The study protocol was conducted following the Helsinki Declaration of 1975, as revised in 1983.

**Fig 1:**
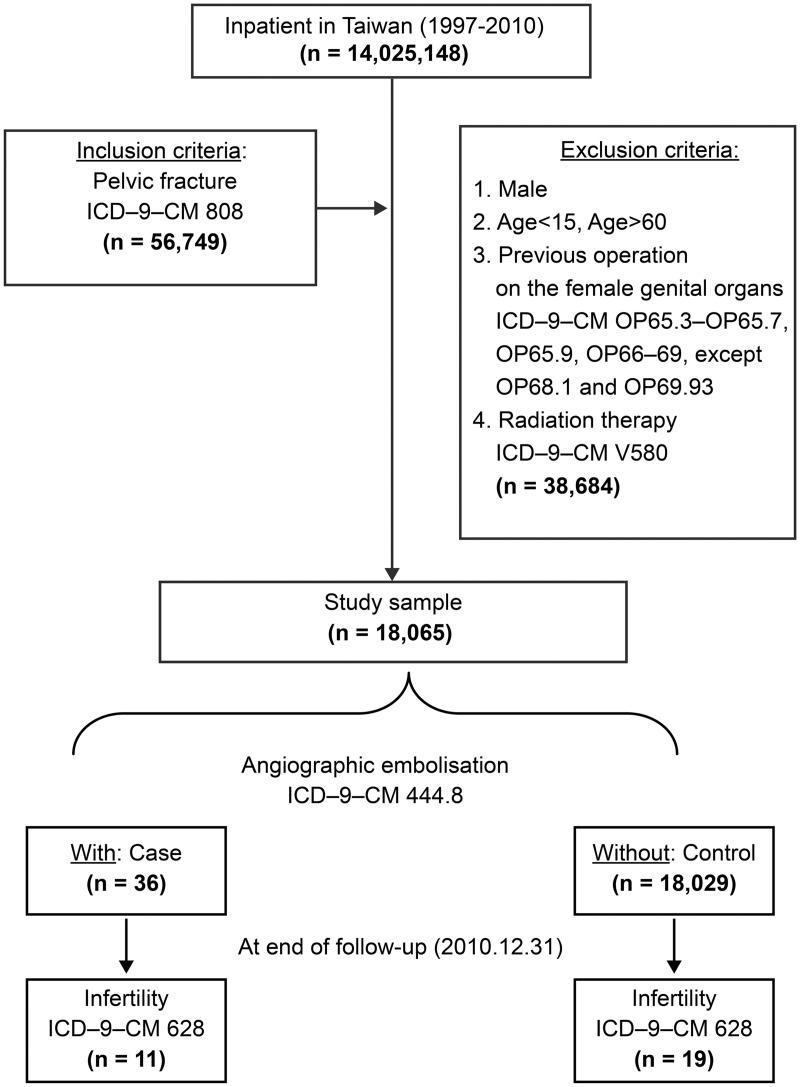
Flowchart of the study sample selection from the National Health Insurance Research Database. ICD–9–CM, International Classification of Diseases, Ninth Revision, Clinical Modification.

We performed a population-based retrospective cohort study to clarify the relationship between pelvic fracture treated with angiographic embolization in female patients and the risk of infertility. Inclusion criterion was the diagnosis of pelvic fracture (ICD–9–CM808) from 1997–2010. Exclusion criteria were male gender, age < 15 or > 60 years, history of prior surgery of the genital organs (ICD–9–CM OP65.3–OP65.7, OP65.9, OP66-69, except OP68.1, OP69.93), and history of radiotherapy (ICD–9–CM V580). Five gynaecologic and obstetric specialists determined the inclusion and exclusion criteria. The index date was set as the date of PAE. A total of 18,065 patients were included. Pelvic fracture patients treated with angiographic embolization (n = 36) were identified and classified as the case (ICD–9–CM 444.8) cohort. The control cohort consisted of pelvic fracture patients without angiographic embolization (n = 18,029). Follow-up period was terminated upon the development of infertility (ICD–9–CM 628), withdrawal from the insurance program, or by December 31, 2010 (Fig 1). Anatomic locations of pelvic fractures were recorded for each patient.

Because a variety of factors can influence infertility, such as age, infection of the genital organs, and severity of concomitant disease [17,18], we considered the following confounding factors: age group (15–35 years, 36–60 years); inflammatory disease of the ovary, fallopian tube, pelvic cellular tissue, and peritoneum (ICD–9–CM 614); inflammatory disease of the uterus (ICD–9–CM 615); inflammatory disease of the cervix, vagina, and vulva (ICD–9–CM 616); endometriosis (ICD–9–CM 617); hypertension (ICD–9–CM 401–5); diabetes mellitus (ICD–9–CM 250); hypertriglyceridemia (ICD–9–CM 275.41); hypercholesterolemia (ICD–9–CM 272.4); coronary artery disease (ICD–9–CM 410–414); history of blood transfusion; and the Injury Severity Score (ISS, ICD–9–CM959.99).

### Statistical analysis

To demonstrate the differences between the case and control cohorts, the count and percentage for category variables (locations of pelvic fracture; age group; inflammatory disease of the ovary, fallopian tube, pelvic cellular tissue, and peritoneum; inflammatory diseases of the uterus; inflammatory disease of the cervix, vagina, and vulva; endometriosis; hypertension; diabetes mellitus; hypertriglyceridemia; hypercholesterolemia; coronary artery disease; blood transfusion; ISS) are presented. The chi-square test or Fisher exact test for categorical variables was used to statistically examine the differences between the two cohorts. The cumulative risk of infertility incidence and demographic-specific and comorbidity-specific infertility incidence for the case and control cohorts were compared using a Cox’s proportional hazards regression model adjusted for potential confounding factors to estimate the hazard ratios (HRs) and 95% confidence intervals (CIs) for the case cohort.

We used SAS 9.3 software (SAS Institute, Cary, NC, USA) to manage and analyse the data. Significance level was set at <0.05, and all tests were two-sided.

## Results

### Patient demographics and follow-up period

A total of 36 and 18,029 patients were included in the case and control cohorts, respectively. The mean age of the case and control cohort was 38.59±15.10 and 37.10±13.45 years, respectively. In the case cohort, the maximum age was 59.70 years and minimum age was 17.60 years. In the control cohort, the maximum age was 60.00 years and minimum age was 15.00 years. Fracture locations and proportion of patients in the two age groups (15–35 years and 36–60 years) were not significantly different between the two cohorts. Proportion of patients with inflammatory disease of the ovary, fallopian tube, pelvic cellular tissue, peritoneum, or with endometriosis was higher in the case cohort than in the control cohort (*p* < 0.001). The proportion of patients with inflammatory disease of the uterus, cervix, vagina and vulva, hypertension, hypertriglyceridemia, hypercholesterolemia, and coronary artery disease was not statistically different between the two cohorts. Eight of 36 patients in the case cohort had a diagnosis of diabetes mellitus versus 561 of 18,029 patients in the control cohort (*p* < 0.001). There was no significant difference in the ISS between the two cohorts (*p =* 0.341). The proportion of patients receiving blood transfusion was higher in the case cohort than in the control cohort (*p* < 0.001; Table 1).

**Table 1:** Patient demographics and comorbidities in the two study cohorts.

Variable	Total		Case		Control		*p*
	n	%	n	%	n	%	
**Total**	18,065		36	0.20	18,029	99.80	
**Pelvic fracture**							0.654
Acetabulum	2,267	12.43	3	8.33	2,264	12.43	
Pubis	7,494	41.07	17	47.22	7,477	41.06	
Others	8,484	46.50	16	44.44	8,468	46.50	
**Age (years)**							0.529
15–35	7,899	43.73	16	44.44	7,883	43.72	
36–60	10,166	56.27	20	55.56	10,146	56.28	
**Inflammatory disease of ovary, fallopian tube, pelvic cellular tissue, and peritoneum**							<0.001
Without	17,889	99.03	30	83.33	17,859	99.06	
With	176	0.97	6	16.67	170	0.94	
**Inflammatory disease of uterus, except cervix**							0.996
Without	18,063	99.99	36	100.00	18,027	99.99	
With	2	0.01	0	0.00	2	0.01	
**Inflammatory disease of cervix, vagina, and vulva**							0.916
Without	18,021	99.76	36	100.00	17,985	99.76	
With	44	0.24	0	0.00	44	0.24	
**Endometriosis**							<0.001
Without	17,891	99.23	32	88.89	17,859	99.26	
With	138	0.77	4	11.11	134	0.74	
**Hypertension**							0.243
Without	17,208	95.26	33	91.67	17,175	95.26	
With	857	4.74	3	8.33	854	4.74	
**Diabetes mellitus**							<0.001
Without	17,222	95.33	25	69.44	17,197	95.39	
With	843	4.67	11	30.56	832	4.61	
**Hyper-triglyceridemia**							0.986
Without	18,058	99.96	36	100.00	18,022	99.96	
With	7	0.04	0	0.00	7	0.04	
**Hyper-cholesterolemia**							0.178
Without	17,967	99.46	35	97.22	17,932	99.46	
With	98	0.54	1	2.78	97	0.54	
**Coronary artery disease**							0.428
Without	17,787	98.46	35	97.22	17,752	98.46	
With	278	1.54	1	2.78	277	1.54	
**Blood Transfusion**							<0.001
Without	17,822	98.65	29	80.56	17,793	98.69	
With	243	1.35	7	19.44	236	1.31	
**ISS ≧ 16**							0.341
Without	16,952	93.84	35	97.22	16,917	93.83	
With	1,113	6.16	1	2.78	1,112	6.17	

*p*-value (category variable: Chi-square/Fisher exact test). ISS = Injury Severity Score

The average follow-up in the case cohort was 3.90±3.56 years; maximum follow-up period was 13.10 years; and minimum follow-up period was 0.01 years. In the control group, average follow-up time was 2.17±3.30 years; maximum follow-up period was 13.94 years; and minimum follow-up period was 0.01 years.

### Risk estimation

The incidence of infertility in the case cohort was 782.70 per 10,000 person–years. In the control cohort, the incidence was 4.85 per 10,000 person–years (Fig 1, Table 2).

**Table 2:** Incidence of subsequent infertility and multivariate Cox proportional hazards regression analysis of the hazard ratios in the two study cohorts.

Variable	Case			Control			Adjusted HR	95%CI	95%CI	*p*
	Event	PYs	Rate (per 10^4^ PYs)	Event	PYs	Rate (per 10^4^ PYs)				
**Total**	11	140.54	782.70	19	39,135.31	4.85	30.676	10.643	70.109	<0.001
**Pelvic fracture**										
Acetabulum	0	5.27	0.00	2	5,008.56	3.99	0.000	-	-	-
Pubis	6	68.94	870.32	11	15,004.74	7.33	22.695	10.130	95.257	<0.001
Others	5	66.32	753.92	6	19,122.00	3.14	42.499	10.285	93.094	<0.001
**Age (years)**										
15–35	9	52.56	1,712.33	14	15,663.03	8.94	49.906	15.177	64.099	**<0.001**
36–60	2	87.98	227.32	5	23,472.28	2.13	23.036	11.023	59.108	**<0.001**
**Inflammatory disease of ovary, fallopian tube, pelvic cellular tissue, and peritoneum**										
Without	5	119.71	417.68	11	38,312.34	2.87	108.104	22.625	516.523	**<0.001**
With	6	20.82	2,881.84	8	822.97	97.21	13.342	7.916	140.436	**<0.001**
**Inflammatory disease of uterus, except cervix**										
Without	11	140.54	782.70	19	39,143.39	4.85	30.675	10.578	70.108	<0.001
With	0	0	-	0	0.92	0.00	-	-	-	-
**Inflammatory disease of cervix, vagina, and vulva**										
Without	11	140.54	782.70	19	39,050.80	4.87	30.675	10.579	70.110	<0.001
With	0	0	-	0	84.51	0.00	-	-	-	-
**Endometriosis**										
Without	7	126.87	551.75	17	38,418.53	4.42	33.127	10.496	49.802	<0.001
With	4	13.67	2,926.12	2	716.77	27.90	27.956	11.257	40.953	<0.001
**Hypertension**										
Without	11	128.55	855.70	19	35,245.76	5.39	30.679	10.648	70.108	<0.001
With	0	11.98	0.00	0	3,889.55	0.00	-	-	-	-
**Diabetes mellitus**										
Without	11	87.73	1,253.85	18	35,605.32	5.06	32.198	21.596	90.845	<0.001
With	0	52.80	0.00	1	3,529.99	2.83	0.000	-	-	-
**Hyper-triglyceridemia**										
Without	11	140.54	782.70	19	39,083.94	4.86	30.676	10.644	70.108	<0.001
With	0	0	-	0	51.37	0.00	-	-	-	-
**Hypercholesterolemia**										
Without	11	138.46	794.45	19	38,588.57	4.92	30.675	10.642	70.110	<0.001
With	0	2.08	0.00	0	546.74	0.00	-	-	-	-
**Coronary artery disease**										
Without	11	132.54	829.94	19	37,842.08	5.02	30.677	10.645	70.111	<0.001
With	0	7.99	0.00	0	1,293.22	0.00	-	-	-	-
**Transfusion**										
Without	11	104.64	1,051.22	19	38,341.41	4.96	30.628	21.630	69.936	<0.001
With	0	35.90	0.00	0	794.00	0.00	-	-	-	-
**Injury Severity Score ≧16**										
Without	11	139.08	790.91	19	36,887.43	5.15	30.675	10.645	70.121	<0.001
With	0	1.46	0.00	0	2,247.88	0.00	-	-	-	-

PYs = person–years; Adjusted HR = adjusted hazard ratio: adjusted for all variables listed in the table; CI = confidence interval; ISS = Injury Severity Score

After adjusting for possible confounding factors, the incidence of infertility in the case cohort was nearly 30.7-fold higher than that in the control cohort (HR = 30.676, 95% CI = 10.643–70.109).

Table 2 shows the estimated HRs of demographic–specific and comorbidity-specific variables for both study cohorts. Patients in both age groups (15–35 years and 36–60 years) had a higher incidence of infertility in the case cohort than in the control cohort (adjusted HR = 49.906 and 23.036, 95% CI = 15.177–64.099 and 11.023–59.108, respectively). Patients without inflammatory disease of the ovary, fallopian tube, pelvic cellular tissue, peritoneum, and endometriosis showed higher risks of developing infertility than patients with these diseases, with adjusted HRs of 108.104 (95% CI = 22.625–516.523) for inflammatory disease of the ovary, fallopian tube, pelvic cellular tissue, and peritoneum and 33.127 (95% CI = 10.496–49.802) for endometriosis.

### Sensitivity analysis

We conducted sensitivity analyses to evaluate the associations between angiographic embolization and the risk of developing infertility according to the duration of follow-up (Table 3). These findings implied that, compared with the control cohort, the case cohort was associated with a significantly higher risk of developing infertility as the follow-up duration was increased. Particularly, patients treated with PAE had a significantly greater incidence of developing infertility when the follow-up period was longer than 2 years; adjusted HRs were 54.496 (95% CI = 15.442–91.176) for follow-up periods of 2–3 years and 38.242 (95% CI = 10.396–80.771) for follow-up periods of >3 years.

**Table 3:** Cox proportional hazards model for the risk of infertility in the study cohorts according to follow-up length.

	Case			Control			Adjusted HR	95%CI	95%CI	*p*
	Event	PYs	Rate (per 10^4^ PYs)	Event	PYs	Rate (per 10^4^ PYs)				
Total	11	140.54	782.70	19	39,135.31	4.85	30.676	10.643	70.109	<0.001
**Time lag (yrs)**										
<1	2	1.95	10,256.41	2	715.35	27.96	31.471	11.755	79.856	0.017
≧1, <2	1	6.36	1,572.33	1	1,929.37	5.18	30.105	16.893	68.432	0.002
≧2, <3	2	15.09	1,325.38	3	2,215.05	13.54	54.496	15.442	91.176	<0.001
≧3	6	117.14	512.21	13	34,275.54	3.79	38.242	10.396	80.771	<0.001

PYs = person–years; Ratio = case rate ÷ control rate; Adjusted HR = adjusted hazard ratio: adjusted for all variables listed in Cox regression table; CI = confidence interval

## Discussion

This is the first large-scale cohort study to evaluate the association between female pelvic fracture treated with PAE and infertility. Based on our results, PAE shows a correlation with the risk of subsequent infertility. After controlling for other important covariates, we found a 30.7-fold increased risk of developing infertility in female pelvic fracture patients treated with angiographic embolization.

Many researchers have evaluated the relationship between PAE for postpartum haemorrhage and fertility. They concluded that embolization is a safe and effective non-surgical treatment that does not affect subsequent fertility [8,9]. Auerbach et al. concluded that selective PAE of the internal iliac branches, including the gluteal arterial branches, appears to be safe in patients with pelvic and acetabular fractures with and without orthopaedic surgical treatment. Nonselective PAE of the internal iliac artery may also be safe when performed unilaterally [19]. One study reported that pelvic trauma did not affect the reproductive function of female patients [20]. However, PAE is associated with other adverse effects; for instance, gluteal muscle necrosis is a well-known complication [6,7].

Potential mechanisms underlying the association between pelvic fractures treated with PAE and infertility in females may include radiation exposure and blood supply. Patients with pelvic fracture treated with PAE will undergo more radiographic exams compared with patients treated without PAE. The actual radiation exposure dose is difficult to measure. The proportion of CT scan in pelvic fracture patients is higher in case cohort than in control cohort (S1 Table). Recently, one study reported that female patients diagnosed with cancer and receiving radiotherapy to the pelvic organs during childhood have a significantly increased risk of infertility [21]. Additionally, impaired blood supply of the female genital organs caused by embolization may also lead to subsequent infertility.

The risk of infertility was lower in patients without pelvic inflammatory diseases than in those with it. This may be due to the fact that women with pelvic inflammatory diseases are already at a higher risk for infertility, and the additional risk after PAE is smaller in magnitude compared to that in the control group.

This study has a number of strengths. First, the sample size is very large, which enhances the statistical power of the data. We used stratified analyses based on the confounding factors of age, transfusion, ISS, comorbidities, and a wide range of demographic characteristics. Second, because we used a nationwide database with a very high coverage rate, almost all patients’ follow-up data are available. Third, the population-based data are representative of the general population in Taiwan.

Our study also has some limitations. First, this is a retrospective cohort study, which has a lower statistical quality. Bias from unknown confounders and errors of primary records may have affected our results, and a well-designed prospective, randomised, controlled study is needed to help establish a causal relationship. Second, the NHIRD does not report information such as menopause, marital status, male-factor infertility of the partner of a patient, usage of oral contraceptives, or smoking, which may have influenced our results. Third, some important clinical information, such as actual ISS, surgical notes, high selective or selective embolization, Gelfoam or steel coil use, radiation exposure time, unilateral or bilateral vessel embolization, number of angioembolizations received, and imaging results were not available because patients included in the NHIRD are anonymous. Fourth, infertility seems to occur in a very small number of patients (11/36 cases and 19/18,029 controls), so we did not choose the control cohort by random sampling. Although the HR of 30.7 was statistically significant, the clinical relevance at such low incidences should be interpreted with caution.

## Conclusions

Female pelvic fracture patients treated with PAE might be associated with a higher risk of developing infertility in Taiwan. Physicians should be aware of the link and inform patients of this risk prior to arterioembolization.

## Supporting information

S1 Table Radiation exposure in the two study cohorts. (DOCX)
