# Supplementary material for: Risk of infertility following pelvic angiographic embolization in female patients with pelvic fractures: A nationwide population-based cohort study in Taiwan
Source: PLoS One. 2017 Dec 1;12(12):e0174733. doi: 10.1371/journal.pone.0174733 (PMC5711024; doi:10.1371/journal.pone.0174733)
Supplement: S1 Table — (DOCX) [file pone.0174733.s001.docx]

**S1 Table 1. Radiation Exposure in the two study cohorts.**

|  | Total |  | Case |  | Control |  |  |
| --- | --- | --- | --- | --- | --- | --- | --- |
| Variable | **n** | **%** | **n** | **%** | **n** | **%** | *p* |
| **CT scan** |  |  |  |  |  |  | 0.208 |
| Without | 15,902 | 88.03 | 30 | 83.33 | 15,872 | 88.04 |  |
| With | 2,163 | 11.97 | 6 | 16.67 | 2,157 | 11.96 |  |
| **Abdomen CT scan** |  |  |  |  |  |  | 0.151 |
| Without | 17,934 | 99.27 | 34 | 94.44 | 17,900 | 99.28 |  |
| With | 131 | 0.73 | 2 | 5.56 | 129 | 0.72 |  |

CT = computed tomography
